# AI-Driven Labor Transformation and Sustainability in Ophthalmology: How to Maintain the Human Perspective in an Increasingly Digital World

**DOI:** 10.22336/rjo.2026.48

**Published:** 2026

**Authors:** Consuela-Mădălina Gheorghe

Artificial intelligence (AI) is gradually entering ophthalmology, changing not only how eye conditions are identified and tracked but also how ophthalmologists work and engage with patients. Innovations that were practically unthinkable ten years ago are now being incorporated into clinical practice. Algorithms can analyze retinal images, identify minute pathological changes, and help doctors diagnose patients. These accomplishments are encouraging. However, they also raise issues that merit as much consideration as the technology itself. What implications will these advancements have for the next generation of ophthalmologists? Will AI actually reduce the increasing demands on medical professionals? Perhaps most crucially, can technical advancements increase the sustainability of ophthalmological treatment without reducing its human component?

Because clinical decisions in ophthalmology often depend on visuals and quantifiable results, the field is especially well-suited to AI applications. Deep learning systems have been shown to diagnose diabetic retinopathy with remarkable accuracy in some of the field’s earliest and most influential studies. Ting et al. (Ting DSW, Cheung CY, Lim G, Tan GSW, Quang ND, et al. Development and Validation of a Deep Learning System for Diabetic Retinopathy and Related Eye Diseases Using Retinal Images From Multiethnic Populations With Diabetes. JAMA. 2017 Dec 12;318(22):2211-2223. doi: 10.1001/jama.2017.18152. PMID: 29234807; PMCID: PMC5820739) showed that automated retinal image analysis applies to multiethnic groups, while Gulshan et al. (Gulshan V, Peng L, Coram M, Stumpe MC, Wu D, et al. Development and Validation of a Deep Learning Algorithm for Detection of Diabetic Retinopathy in Retinal Fundus Photographs. JAMA. 2016 Dec 13;316(22):2402-2410. doi: 10.1001/jama.2016.17216. PMID: 27898976) provided compelling evidence of its diagnostic potential. De Fauw et al. (De Fauw J, Ledsam JR, Romera-Paredes B, Nikolov S, Tomasev N, et al. Clinically applicable deep learning for diagnosis and referral in retinal disease. Nat Med. 2018 Sep;24(9):1342-1350. doi: 10.1038/s41591-018-0107-6. Epub 2018 Aug 13. PMID: 30104768) then showed how AI may help interpret optical coherence tomography images and inform referral decisions. Abràmoff et al. (Abràmoff MD, Lavin PT, Birch M, Shah N, Folk JC. Pivotal trial of an autonomous AI-based diagnostic system for detection of diabetic retinopathy in primary care offices. NPJ Digit Med. 2018 Aug 28;1:39. doi: 10.1038/s41746-018-0040-6. PMID: 31304320; PMCID: PMC6550188) also evaluated an autonomous AI system for detecting diabetic retinopathy in primary care, another noteworthy milestone. Taken together, these results suggest that direct specialist evaluation is no longer necessary for several diagnostic tasks. They also suggest an approach to eye care that makes early detection more accessible, especially for patients who might otherwise have to travel long distances or wait long periods for specialized services.

Nonetheless, the ability to identify illness should not be confused with the ability to provide patient care. Although a retinal scan can show pathological changes, it provides little information about the individual. It cannot describe how a person’s independence is affected by visual impairment, whether a patient fears losing their sight, or how a diagnosis may affect their family and career. These are characteristics of medicine that are difficult to quantify. Ophthalmologists do much more than interpret images. They analyze treatment options, clarify uncertainties, put findings in clinical context, and help patients make decisions with long-term effects. Ultimately, AI may remind us of the importance of clinical judgment, empathy, and communication rather than diminish doctors’ role.

Given the current pressures on ophthalmological services, this becomes even more crucial. Diabetes continues to place a significant strain on healthcare systems; populations are aging, and long-term monitoring is necessary for chronic illnesses, including glaucoma and age-related macular degeneration. At the same time, many healthcare systems struggle to provide prompt access to specialist consultations. In these situations, AI-assisted screening offers a useful opportunity to reevaluate how clinicians use their time. Ophthalmologists may be able to focus more on complex diagnoses, treatment planning, and patients who require closer clinical supervision if dependable automated methods can detect patients who need urgent assessment while safely removing low-risk cases from unnecessary referrals.

However, improved working conditions are not always correlated with increased technical efficiency. Innovation can sometimes create more problems than it solves, as anyone familiar with implementing new digital systems in healthcare knows. Digital platforms produce data that must be analyzed and recorded, automated diagnostic instruments need supervision, and ambiguous results need confirmation. Doctors may spend more time managing alerts, reviewing algorithmic recommendations, or navigating increasingly complex workflows than analyzing images. The crucial question is whether AI actually improves the daily lives of patients and medical personnel, rather than simply enabling more tests in less time. Instead of adding yet another layer of technological responsibility to an already demanding profession, a good implementation should give ophthalmologists more time to practice medicine.

Medical education also faces important implications. Becoming a skilled ophthalmologist takes much more than learning theory. Years of observation, frequent clinical exposure, real-world experience, and the steady growth of diagnostic confidence are all necessary. Younger doctors might have fewer opportunities to develop these skills independently if automated systems handle routine image interpretation. This does not mean AI should be excluded from medical education. Instead, aspiring ophthalmologists must understand how these systems work, recognize their limitations, and critically evaluate their suggestions. Ensuring that technology supports rather than gradually replaces clinical reasoning will be a challenge. The development of autonomous diagnostic systems challenges us to reevaluate the connection between artificial intelligence and medical knowledge, as Keane and Topol (Keane PA, Topol EJ. With an eye to AI and autonomous diagnosis. NPJ Digit Med. 2018 Aug 28;1:40. doi: 10.1038/s41746-018-0048-y. PMID: 31304321; PMCID: PMC6550235) explain.

Sustainability adds a crucial new perspective to this conversation. Because healthcare resources are scarce, ophthalmological services must meet rising demand without sacrificing care quality. From an economic perspective, AI may help distribute resources more effectively by improving patient triage, enabling earlier diagnosis, and reducing unnecessary specialist appointments. However, organizations should not take these advantages for granted. Buying software is just the first step. Healthcare organizations must also consider equipment, maintenance, data security, cybersecurity, technical support, and professional training. When implemented across an entire healthcare network, a solution that seems cost-effective during a trial study could end up costing far more. Therefore, its worth should be evaluated not only in terms of immediate productivity but also in terms of its long-term consequences on healthcare costs, staff workload, and patient outcomes.

Similar consideration should be given to environmental sustainability. Remote screening and more efficient referral systems may reduce needless travel, recurrent hospital stays, and some of the resources involved in traditional healthcare delivery. These changes can significantly benefit patients who live far from specialized facilities. But it is important to consider AI’s environmental consequences. Data processing, digital storage, computing infrastructure, and manufacturing and replacing electronic devices require resources. Therefore, it is oversimplified to assume that digital healthcare is inherently more environmentally friendly than traditional treatment. A responsible approach should assess whether the advantages outweigh the resources used and account for the environmental impact of the entire technical process.

AI’s promise to increase access to ophthalmological treatment is perhaps the strongest justification for the technology. Automated retinal screening could help identify vision-threatening conditions sooner in underprivileged communities and rural areas where access to specialized services may be limited. For some patients, early detection could be the difference between maintaining functional eyesight and suffering irreparable vision loss. However, technology alone cannot resolve healthcare disparities. Screening is only beneficial when patients can receive the necessary follow-up tests and care. Even the most advanced algorithm may have little impact in practice without dependable infrastructure, qualified staff, and efficient referral channels. Additionally, wealthier institutions may adopt new technology faster, widening the gap between well-resourced and underserved communities. The World Health Organization has highlighted the importance of autonomy, accountability, openness, and equity in healthcare AI (World Health Organization. Ethics and Governance of Artificial Intelligence for Health: WHO Guidance. Geneva: World Health Organization; 2021). When deciding how to incorporate these technologies into ophthalmological practice, these concepts should remain crucial.

Ultimately, AI will likely reshape ophthalmology, but its impact will extend far beyond redistributing diagnostic duties. It will affect how doctors receive training, how clinical duties are distributed, how healthcare facilities allocate resources, and how patients receive care. Rather than prompting unquestioned zeal or opposition, these adjustments should encourage introspection. Medical advancement has always been shaped by technology, but its usefulness has always depended on the goals it serves and the views of its users.

The coming years may offer ophthalmologists a chance to reevaluate what should receive most of their professional attention. If AI can reliably handle repetitive tasks, physicians may have more time to discuss challenging diagnoses, help patients make treatment decisions, and provide the assurance often required when vision is at risk. Such a result would be progress not only because healthcare is becoming faster but also because it is becoming more focused on individual needs.

Therefore, the number of activities computers can complete on their own, or the degree to which artificial intelligence can mimic the judgments of skilled physicians, should not gauge the future of ophthalmology. Whether these technologies enable ophthalmologists to provide better care while maintaining their professional knowledge, promoting their well-being, and using healthcare resources responsibly will be a more significant metric.

After all, the ultimate goal of ophthalmology is to safeguard a vital aspect of human existence—the capacity to see, maintain independence, and interact with the outside world—rather than only diagnosing illness. Only when it helps doctors achieve that goal more successfully while maintaining the human link that lends medicine much of its significance will artificial intelligence have lived up to its promise.

**Senior Lecturer Consuela-Mădălina Gheorghe, PhD, Philologist, Certified translator**

